# The potential immunomodulatory role of the gut microbiota in the pathogenesis of asthma: an in vitro study

**DOI:** 10.1038/s41598-023-47003-0

**Published:** 2023-11-13

**Authors:** Paulina Kleniewska, Paulina Natalia Kopa-Stojak, Arkadiusz Hoffmann, Rafał Pawliczak

**Affiliations:** 1 Department of Immunopathology, Faculty of Medicine, Medical University of Lodz, Zeligowskiego 7/9, 90-752 Lodz, Poland; 2 Department of Immunopathology, Faculty of Medicine, Medical University of Lodz, Zeligowskiego 7/9, bldg 2, Rm 177, 90-752 Lodz, Poland

**Keywords:** Diseases, Medical research

## Abstract

The aim of this study was to investigate the influence of *Bacteroides vulgatus *(*BV*), *Clostridium perfringens *(*CP*), *Parabacteroides distasonis *(*PD*) and *Ruminococcus albus *(*RA*) lysates on secretion of selected cytokines by PBMC, MDM and HT-29 cells, as well as to determine the potential mechanisms of their action in the development of asthma. Enzyme-linked immunosorbent assays were used to analyze the effect of BV, CP, PD and RA lysates on the secretion of IL-1β, IL-6, IL-10 and TNF-α by human PBMC, MDM and HT-29 cells. BV and CP lysates significantly lowered IL-1β secretion by MDM vs. control (p < 0.05 and p < 0.001 respectively) but only at a dose of 400 µg lysate. The secretions of IL-6 by PBMC and MDM were elevated significantly above control values (p < 0.05) after administration of CP and PD lysates. BV, CP and PD lysates (100 µg) significantly increased IL-10 secretion by PBMC vs. control (p < 0.05). CP, PD and RA lysates (400 µg) significantly increased IL-10 secretion by MDM vs. control (p < 0.001). BV lysate (400 µg) also significantly increased IL-10 secretion by MDM as compared to control (p < 0.05). In PBMC and MDM, the production levels of the anti-inflammatory cytokine were increased by all the bacterial lysates used in a dose-dependent manner.

## Introduction

Asthma is a lung disease associated with chronic inflammation and airway obturation^1^. The most common asthma symptoms are wheezing, coughing, breath shortness and chest tightness^2,3^. This disease affected about 262 million people in 2019^4–6^. In 2017, the annual costs of asthma treatment were estimated in Europe at about 1900 USD per patient^7^. Asthma pathology is a multifactorial process associated with chronic airway inflammation which leads to the airflow limitation and also causes bronchial hyper-reactivity^8^. The main risk factors for developing asthma include genetic predisposition and environmental factors, viral respiratory infections in childhood or diet^9^.

Gut microbiota has many functions, including: vitamins production, protection from pathogens and enhancement immune response^10,11^. Several studies confirm that in the first year of a child’s life, microbiological stimulation has a significant impact on the maturation of lymph tissue occurring within the gastrointestinal tract^12^. Experiments on animal models have proved that stimulation with intestinal bacteria has a significant impact on the diversity of antibodies present in gastrointestinal tract immediately after birth^13,14^. Studies on GF mice have shown that the development of oral tolerance occurs if the intestinal flora is re-colonized by *Bifidobacterium*, but no later than in the neonatal period. In addition, bacteria colonize the gut and induce a pronounced immune response to IgA production^15,16^.

Dysbiosis in early life may predispose to the development of many respiratory diseases, such as asthma, which is associated with the impact of gut microflora on immune system maturation^17^. In fecal samples of newborns with high asthma development risk reduced amount of *Lachnospira*, *Veillonella*, *Faecalibacterium*, and *Rothia* strains was observed^18^. In addition, an increased risk of atopy and asthma was also observed in neonates with an increase in the volume of *Streptococcus* and *Bacteroides* spp. and a decrease in the number of *Bifidobacterium* spp. and *Ruminococcus gnavus* in fecal samples^19^. Moreover, lower abundance of *Bifidobacteria*, *Akkermansia* and *Faecalibacterium* spp. in the intestines predisposes to the higher risk of atopy and asthma development in children^20^. Therefore further intensive research is needed to determine how individual intestinal bacteria may influence the development of asthma, as well as to determine potential mechanisms of their action. The aim of this study was to investigate the influence of *Bacteroides vulgatus*, *Clostridium perfringens*, *Parabacteroides distasonis* and *Ruminococcus albus* lysates on the secretion of selected cytokines by PBMC, MDM and HT-29 cells, as well as to determine the potential mechanisms of action of these species of intestinal microflora in the development of asthma.

## Materials and methods

### Bacterial strains

*Ruminococcus albus* (ATCC 27210, LGC Standards, Teddington, UK) was harvested in ATCC 158 RGCA Medium; *Clostridium perfringens* (ATCC 13124, LGC Standards, Teddington, UK) was cultivated in ATCC 2107 Modified Reinforced Clostridial medium (LGC Standards, Teddington, UK), *Bacteroides vulgatus* (ATCC 8482, LGC Standards, Teddington, UK) was harvested in ATCC 2107 Modified Reinforced Clostridial Medium and *Parabacteroides distasonis* (ATCC 8503, LGC Standards, Teddington, UK) was cultivated in ATCC 1490 Modified Chopped Meat medium in 37 °C in an anaerobic conditions. As a selected medium, ATCC 260 Trypticase soy agar with 5% sheep blood was used.

### Human cell lines

Human peripheral blood mononuclear cells (PBMC; SigmaAldrich, Saint Louis, MO, USA) were harvested in Mononuclear Cell Medium (SigmaAldrich, Saint Louis, MO, USA) and human HT-29 cells (LGC Standards, Teddington, UK) were cultivated in McCoy’a 5A (ATCC 30-2007) and fetal bovine serum (500 ml, ATCC 30-2020). Human monocytes were isolated from PBMC (SigmaAldrich, Saint Louis, MO, USA) by incubation for 2 h in RPMI-1640 medium (SigmaAldrich, Saint Louis, MO, USA). Remaining cells were removed from the ones that adhered to the culture flask surface by washing (three times), which allowed monocytes to adhere to the cells that stayed on the surface of the flask. The remaining cells were removed in warm RPMI-1640 medium (SigmaAldrich, Saint Louis, MO, USA). Human monocytes were then harvested in RPMI-1640 medium (SigmaAldrich, Saint Louis, MO, USA) supplemented with 10% FBS (SigmaAldrich, Saint Louis, MO, USA). Each cell line was harvested in T75 flasks in standard conditions (37 °C, 5% CO_2_, 90% humidity). In the experiment, combination of antibiotics was used (SigmaAldrich, #P4333, Penicilin/Streptomycin; Lonza #195266).

### Preparation of *Ruminococcus albus*, *Clostridium perfringens*, *Bacteroides vulgatus* and *Parabacteroides distasonis*

Intestinal microflora cultures of *Ruminococcus albus*, *Clostridium perfringens*, *Bacteroides vulgatus* and *Parabacteroides distasonis* were transferred into collection tubes and centrifuged for 5 min, 10,000 rpm at 4 °C. After bacterial cell pellet suspension in 1 ml of distilled water bacterial lysates were prepared by ultrasonic disintegration of cells structure. Samples were then centrifuged at the same conditions and the obtained supernatants were transferred into new collection tubes and stored at − 20 °C.

### Stimulation of human PBMC, human MDM and HT-29 cells by *Ruminococcus albus*, *Clostridium perfringens*, *Bacteroides vulgatus* and *Parabacteroides distasonis* lysates

Human PBMC were seeded on a plate at 2 × 10^6^ cells/well in a 6-well. Human MDM and HT-29 cells were seeded at 0.5 × 10^6^ cells/well. Then, the cell lines were stimulated with *Ruminococcus albus*, *Clostriduim perfringens*, *Bacteroides vulgatus* and *Parabacteroides distasonis* lysates (two different concentrations: 100 µg and 400 µg) for 24 h. Negative and positive control were the cells incubated respectively with fresh culture medium and with 25 µg/ml dexamethasone. After 24-h stimulation of PBMC, monocytes and HT-29 cells with intestinal microbiota lysates, the samples were centrifuged for 5 min at 1500 rpm and 4 °C. The obtained supernatants were transferred into new collection tubes and stored at − 20 °C.

### Analysis of selected secretion of cytokines by PBMC, human MDM and HT-29 cells after incubation with intestinal microbiota lysates

To analyze selected secretion of cytokines by PBMC, MDM and HT-29 cells after incubation with *Ruminococcus albus*, *Clostridium perfringens, Bacteroides vulgatus* and *Parabacteroides distasonis* lysates, enzyme-linked immunosorbent assays were performed. Commercially available ELISA kits from SigmaAldrich (Sain Louis, MO, USA) were used. For IL-1β, IL-6, IL-10 and TNF-α analysis, 100 µl of standards and 100 µl of each sample was added into 96-well ELISA plates in duplicates. The plates were incubated for 2.5 h (ambient temperature, gentle shaking). After the incubation, the plates were washed 4 times with 300 µl/well of Wash solution. Then, 100 µl of detection antibody was added into each well and incubated for 1 h (room temperature, gentle shaking). After the incubation, the plates were washed again 4 times with 300 µl/well of wash solution. Then, 100 µl of Streptavidin solution was added into each well and incubated for 45 min (room temperature, gentle shaking). Next, the plates were washed again 4 times with 300 µl/well of wash solution. Then, 100 µl of Substrate reagent was added to each well and incubated for 30 min at room temperature, in the dark, with gentle shaking. Immediately after adding 50 µl of Stop solution to the plates, absorbance at 450 nm was read on the microplate reader.

### Statistical analysis

Results were presented as MEAN ± SEM. The statistical analysis was performed using one-way ANOVA followed by Tukey multiple range post hoc test and Dunnett’s method. The p < 0.05 was considered as statistically significant.

## Results

### Evaluation of IL-1β, IL-6, IL-10 and TNF-α concentrations

**Figure 1:**
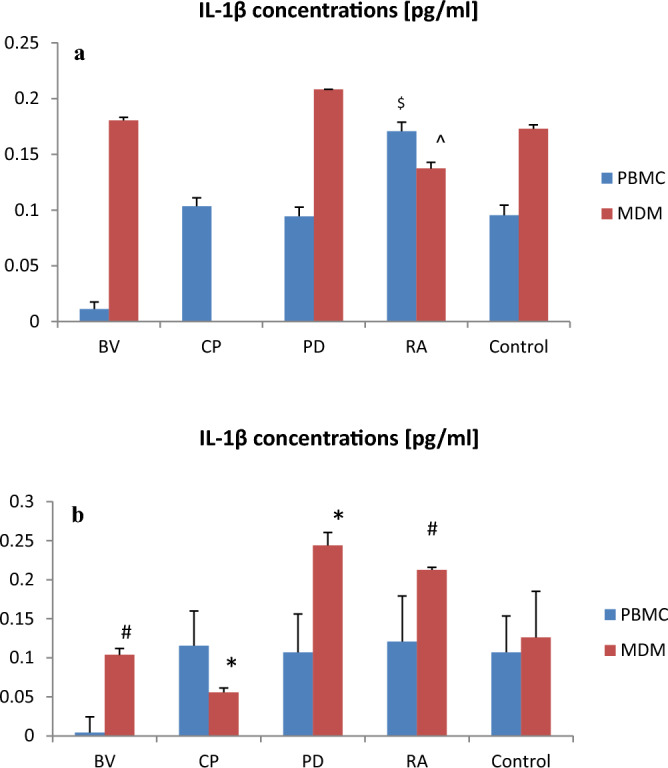
The effect of bacterial lysates at a dose of 100 µg (**a**) and at a dose of 400 µg (**b**) on IL-1β secretion by human peripheral blood mononuclear cells (PBMC) and human monocyte-derived macrophages (MDM). Tukey’s Test: *p < 0.001 vs. control group; ^#^p < 0.05 vs. control group; ^$^p < 0.05 vs. BV PBMC; ^p < 0.05 vs. PD MDM. *BV Bacteroides vulgatus*, *CP Clostridium perfringens*, *PD Parabacteroides distasonis*, *RA Ruminococcus albus*.

*Bacteroides vulgatus* and *Clostridium perfringens* lysates significantly lowered IL-1β secretion by MDM as compared to control (respectively p < 0.05; p < 0.001) but only at a dose of 400 µg lysate (Fig. 1). *Ruminococcus albus* lysates slightly reduced the IL-1β concentration by MDM vs. control (100 µg of lysate).

**Figure 2:**
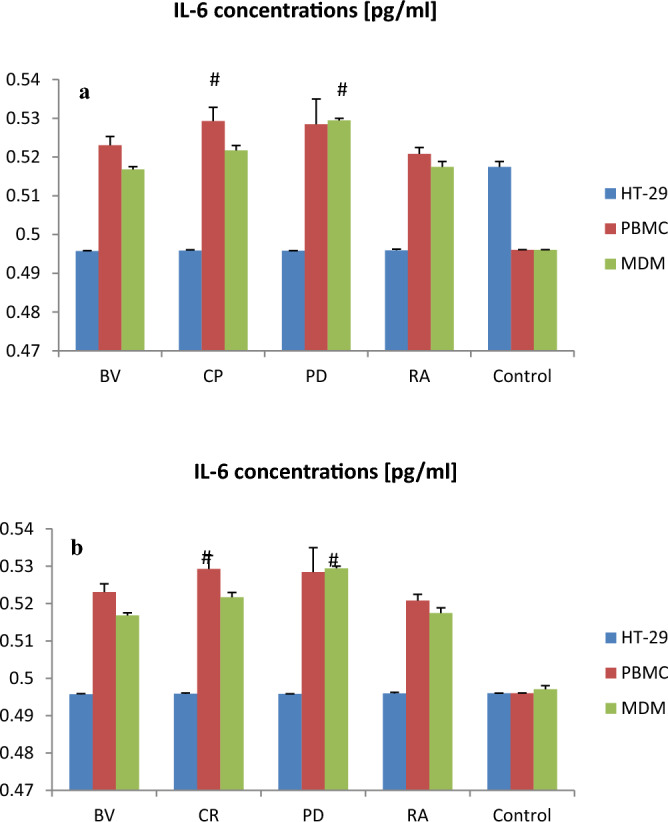
The effect of bacterial lysates at a dose of 100 µg (**a**) and at a dose of 400 µg (**b**) on IL-6 secretion by human intestinal epithelial cells (HT-29), human peripheral blood mononuclear cells (PBMC) and human monocyte-derived macrophages (MDM). Data is shown as mean ± S.E.M; Tukey’s Test: ^#^p < 0.05 vs. control group; ^p < 0.05 vs. HT-29. *BV Bacteroides vulgatus*, *CP Clostridium perfringens*, *PD Parabacteroides distasonis*, *RA Ruminococcus albus*.

**Figure 3:**
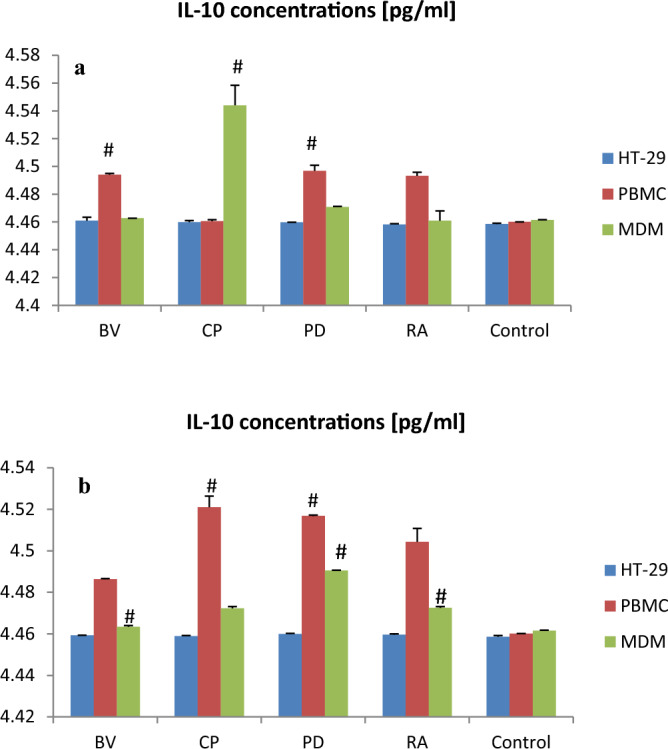
The effect of bacterial lysates at a dose of 100 µg (**a**) and at a dose of 400 µg (**b**) on IL-10 secretion by human intestinal epithelial cells (HT-29), human peripheral blood mononuclear cells (PBMC) and human monocyte-derived macrophages (MDM). Data is shown as mean ± S.E.M; Dunnett’s Method: *^#^*p* < *0.05* vs. control group. *BV Bacteroides vulgatus*, *CP Clostridium perfringens*, *PD Parabacteroides distasonis*, *RA Ruminococcus albus*.

*Clostridium perfringens* lysate significantly increased IL-6 secretion by PBMC as compared to control (p < 0.05; Fig. 2). *Parabacteroides distasonis* lysate significantly increased IL-6 secretion by MDM vs. control (p < 0.05). All other lysates also increased IL-6 secretion by PBMC and MDM as compared to control but without statistical significance (Fig. 2). In contrast, bacterial lysates (100 µg) reduced the IL-6 secretion by HT-29; however, these changes were not significant. *Bacteroides vulgatus, Clostridium perfringens and Parabacteroides distasonis* lysates (100 µg) significantly increased IL-10 secretion by PBMC as compared to control (p < 0.05). *Clostridium perfringens and Parabacteroides distasonis* lysates (400 µg) significantly increased IL-10 secretion by PBMC and MDM vs. control (p < 0.05). *Clostridium perfringens*, *Parabacteroides distasonis* and *Ruminococcus albus* lysates (400 µg) significantly increased IL-10 secretion by MDM as compared to control (p < 0.001). *Bacteroides vulgatus* lysate (400 µg) also significantly increased IL-10 secretion by MDM vs. control (p < 0.05; Fig. 3).

**Figure 4:**
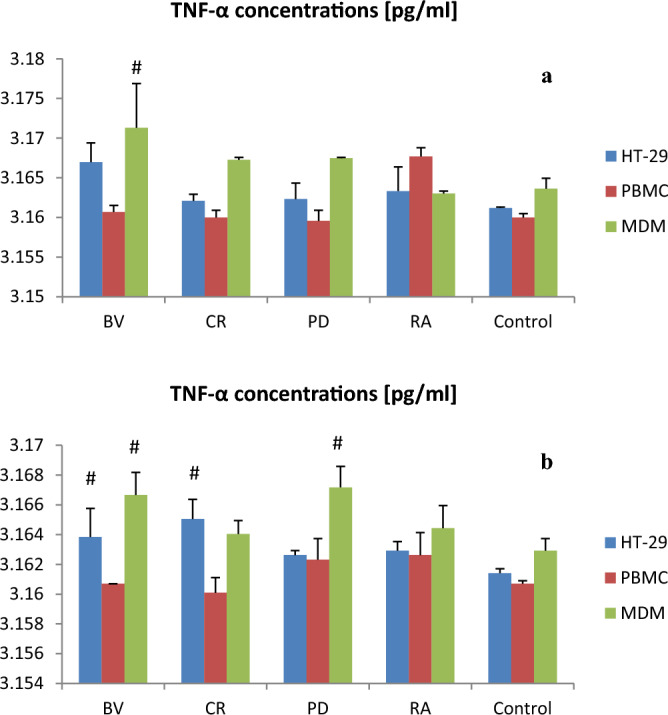
The effect of bacterial lysates at a dose of 100 µg (**a**) and at a dose of 400 µg (**b**) on TNF-α secretion by human intestinal epithelial cells (HT-29), human peripheral blood mononuclear cells (PBMC) and human monocyte-derived macrophages (MDM). Data is shown as mean ± S.E.M; Dunnett’s Method: ^*#*^*p* < *0.05* vs. control group. *BV Bacteroides vulgatus*, *CP Clostridium perfringens*, *PD Parabacteroides distasonis*, *RA Ruminococcus albus*.

*Bacteroides vulgatus* lysate (100 µg) significantly increased TNF-α secretion by MDM as compared to control (p < 0.05). Moreover *Bacteroides vulgatus* lysate (400 µg) significantly increased TNF-α secretion by HT-29 and MDM as compared to control (p < 0.05). *Parabacteroides distasonis* lysates (400 µg) significantly increased TNF-α secretion by MDM vs. control (p < 0.05; Fig. 4).

## Discussion

The role of chronic inflammation and airway remodeling in the pathogenesis of bronchial hyperresponsiveness may change during the clinical course of asthma. In the initial period, inflammation plays the dominant role, then bronchial hyperresponsiveness depends on tissue remodeling. Moreover, as the disease progresses, bronchial structural cells play an increasingly important role in the amplification of the inflammation by releasing inflammatory mediators. They are an important source of chemokines, cytokines and other inflammatory mediators maintaining the inflammatory process. The bronchial epithelium plays an active role in the pathogenesis of asthma. Interaction with environmental factors leads to the release of thymic stromal lymphopoietin and interleukins: 25 and 33. The epithelium is a rich source of pro-inflammatory cytokines such as TNF-α, chemokines, growth factors and interferons. In asthma patients the infectious agent induces lower production of interferon-γ by inflammatory cells and lower production of interferon P and X by respiratory epithelial cells, which makes it difficult to generate effective defense reaction against viruses.

The role of gut microbiota is still underestimated. Our present findings indicate that *B. vulgatus* and *C. perfringens* lysates at a dose of 400 µg significantly lowered IL-1β secretion by MDM vs. control. Whereas, *R. albus* lysates at a dose of 100 µg only slightly reduced the IL-1β concentration by MDM compared to control.

The role of *Bacteroides* in the course of various diseases has been described in many studies, including alleviating obesity^21^, preventing atherosclerosis^22^, protecting against colitis^23^ and inhibiting cancer^24^. Unfortunately, there are no similar studies linking BV and asthma.

The results of the Liu et al. study^25^ showed that administration of *B. vulgatus* Bv46 reduces the expression of interleukins 1β, 6 and TNF-α in the colon in dextran sodium sulfate-induced colitis in vivo and diminishes the secretion of interleukins- 1β, 6 and TNF-α in macrophages stimulated by LPS in vitro. What's more BV downregulates Ccl19, Cd19, Cd22, Cd40 and Cxcr5 genes in the colon of mice, which are mainly involved in the regulation of B cell responses. Additionally, BV in combination with *B. dorei* (BD) reduces the concentration of lipopolysaccharides in serum and feces, prevents cytokine induction and inhibits atherosclerosis. Scientists have shown that treatment with live BV and BD can help prevent coronary artery disease^22^.

However, some studies in other experimental models report increased IL-1β secretion. Fasina’s research indicated that after the CP challenge, the expression level of interleukin 1β was upregulated^26^. Another study also demonstrated that high dose of CP significantly increased pro-inflammatory factors such as interleukins 1β, 6 and TNF-α^27^. Tang et al.^28^ report that CP challenge can cause an upregulation on pro-inflammatory mediator genes including TNF-α and interleukins: 1β, 8 in the intestine of broilers.

Our results suggest that *C. perfringens* lysates significantly increased IL-6 secretion by PBMC and by MDM compared to control. All other lysates also increased IL-6 secretion by these cells; however, these changes were not significant. In contrast, bacterial lysates caused a statistically insignificant decrease in interleukin 6 concentration in HT-29 cells.

Guo et al. described that CP infection elevated levels of interleukins- 6, 8^29^. In the next study^30^, a homopolysaccharide fraction isolated from culture broth of PD exerted an immunostimulatory effect by promoting the secretion of interleukins-1β, 6 and TNF-α.

However, another studies report significant decrease in IL-6 secretion. Some authors have shown that PD has anti-inflammatory properties^31^. In 2021, scientists published data on the protective role of selected strains of *B. vulgates* against colitis caused by dextran sodium sulfate (DSS). Among the tested strains, *B. vulgatus* 7K1 turned out to be the most effective in significantly inhibiting the increase in IL-6 and TNF-α concentrations and increasing IL-10 concentrations to the level found in the control group^32^.

This paper shows that *B. vulgatus, C. perfringens and P. distasonis* lysates (100 µg) significantly increased IL-10 secretion by PBMC compared to control. CP and PD lysates (400 µg) significantly increased IL-10 secretion by PBMC and MDM compared to control. *C. perfringens*, *P. distasonis* and *R. albus* lysates (400 µg) significantly increased IL-10 secretion by MDM compared to control. *B. vulgatus* lysate (400 µg) also significantly increased IL-10 secretion by MDM compared to control.

Similar results, that PD supplementation leads to an increase in IL-10 levels were reported by Kverka et al.^33^. The authors proved that PD, has the ability to reduce the intestinal inflammation by inducing the anti-inflammatory cytokine IL-10 and suppressing the secretion of inflammatory interleukins- 6 and 17. The results of recent studies confirm that *B. vulgatus* FTJS7K1 supplementation can significantly increase the level of mRNA of the anti-inflammatory factor IL-10 compared to the LPS groups. This bacterial strain reduces acute inflammation and intestinal injury in mice by modulating the gut microbial community. The authors showed that the genes responsible for the secretion of SCFA are responsible for the anti-inflammatory effect^34^.

Probiotic strains of *Bifidobacteria* in BFM may interfere with the intestinal inflammatory response. The authors described the effect of probiotic strains on IL-10 secretion by peripheral blood mononuclear cells and IL-8 production by intestinal epithelial cells. *B. vulgatus*, *B. fragilis* and *B. thetaiotaomicron* reduced the transcription of poly(I:C)-induced inflammatory genes^35^.

Our results show that *B. vulgatus* lysate (100 µg) significantly increased TNF-α secretion by MDM compared to control. Moreover, *B. vulgatus* lysate at a dose of 400 µg significantly increased TNF-α secretion by HT-29 and MDM compared to control. PD lysates (400 µg) also significantly increased TNF-alpha secretion by MDM as compared to control.

In 2022, Chamarande et al.^36^ published data on the properties of *P. distasonis* strains. In PBMC, the production levels of the proinflammatory cytokines, such as interleukins 1β, 6 and TNF-α were increased by almost all the PD strains tested. Moreover, the PD treatment during *E. coli* LPS-induced inflammation did not reduce the proinflammatory cytokine production of PBMC. These results, contrary to HT-29 results, suggest a proinflammatory response of the immune system cells due to *P. distasonis* stimulation, although with strain variability.

However, some studies report a decrease in TNF-α secretion. The authors described that oral treatment with *B. vulgatus* might reduce dysbiosis of the colonic microbiota by reducing LPS/TLR-4/p-NF-κB signaling pathway in the colon and serum TNF-α to attenuate lumbar bone loss in ovariectomized mice. In the same study, no differences were observed for interleukins 1β, 6 and 8^37^.

Papers published more than a decade ago have already indicated a relationship between the microbiome and the asthma symptoms occurring. Correlation between *Bacteroides* and IgE/IgG immune response in allergic children was investigated by Fukuda et al.^38^. A higher IgG titer to *B. vulgaris* was found in the children with allergic symptoms. Kirjavainen et al.^39^ indicated that in infants intolerant to extensively hydrolyzed whey formula, serum total IgE correlates with faecal *Bacteroides* counts at 4–6 months of age. Another study^40^ also confirms that the faecal counts of *Bacteroides* at age 5 years correlate positively with the serum IgE concentration. Sudo et al.^41^ proved that kanamycin-induced elevation of the serum IgE levels in mice was improved by the inoculation with BV. An increased ratio of *Bacteroides fragilis* to *Bifidobacterium* bacteria in the faeces of adults allergic to pollen during the pollen season has been demonstrated. This increase was prevented by *Bifidobacterium* administration. Scientists, using PBMC of patients with pollen allergy also showed that *B. fragilis* strains induced more Th2 cytokines but fewer Th1 cytokines compared with *Bifidobacterium* strains^42^.

An interesting study conducted in the U.S. suggests that the "high risk group" of developing allergic reactions was characterized by a relatively lower number of *Bifidobacterium*, *Akmermansia* and *Faecalibacterium* and a higher number of *Candida* and *Rhodotorula*^20^. Similar publications proved that patients diagnosed with severe asthma are characterized by a significantly lower number of *Bifidobacterium* bacteria in the intestinal flora^43^. It is worth noting that the formation of microflora is a gradual phenomenon. Thus, it is possible to influence its development through a proper diet or probiotics/synbiotics supplementation. A full understanding of the “gut-lung” axis may be helpful in the asthma treating and preventing. The mechanism by which these two systems can affect each other is not well understood, however, inflammation that has been initiated in the intestines may result in the lungs inflammation as a result of the destructive effects of the over-stimulated immune system.

To investigate new, potentially therapeutic applications of probiotic bacteria in relieving asthma symptoms, researchers evaluated the effects of long-term use of *Bifidobacterium breve* M-16 V and *Lactobacillus rhamnosus* NutRes1 with glucocorticoid therapy as a reference medicine. The study showed that *L. rhamnosus* is as effective as budesonide in alleviating the inflammatory response and reducing airway resistance in diseased animals. In addition, both probiotic microorganisms successfully alleviated chronic allergic inflammation by weakening the total number of inflammatory cells and individual BAL fluid cell counts^44^. Moreover, studies on animal models have confirmed the adverse role of antibiotics as one of the reasons for the increased incidence of asthma, which is consistent with the “hygiene hypothesis”^45–47^.

It turns out that, unfortunately, there are not enough research projects with a similar methodology to draw clear conclusions. Without systematizing research techniques, conclusions from experiments will remain ambiguous. Supplementing the information on a detailed therapy regimen and the selection of specific strains for specific groups of patients is necessary to fully use the potential of microorganisms in the treatment of asthma. The effectiveness of selected microorganisms requires a detailed knowledge of the entire human microbiome, as well as checking, among others: the impact of modified bacteria on the microflora in the long term. Not only the effectiveness, but also the safety of the species/strains used should be carefully assessed, which, may represent a unique opportunity for many patients with bronchial asthma in the future.

## Abbreviations

BV: *Bacteroides vulgatus*
CP: *Clostridium perfringens*
MDM: Human monocyte-derived macrophages
PD: *Parabacteroides distasonis*
PBMC: Peripheral blood mononuclear cells
RA: *Ruminococcus albus*
